# Camel filariasis (*Dipetalonema evansi*) and its association with clinical balanoposthitis with reference to prominent changes in clinical findings, serum testosterone, semen analysis, and testicular histopathology

**DOI:** 10.1186/s12917-023-03844-5

**Published:** 2024-01-03

**Authors:** Arafat Khalphallah, Taher Al-Daek, Mahmoud Abdelhamid, Enas Elmeligy, Sayed Fathi El-Hawari, Khaled A. Khesruf, Heba A. Nasr, Ragab H. Mohamed

**Affiliations:** 1 Division of Internal Medicine, Department of Animal Medicine, Faculty of Veterinary Medicine, Assiut University, Assiut, 71526 Egypt; 2 Faculty of Veterinary Medicine, Omar Al-Mukhtar University, Al-bayda, 919 Libya; 3 Department of Parasitology, Faculty of Veterinary Medicine, Aswan University, Aswan, 81528 Egypt; 4 Veterinary Teaching Hospital, Faculty of Veterinary Medicine, Assiut University, Assiut, 71526 Egypt; 5 Department of clinical studies, Collage of Veterinary Medicine, King Faisal University, Al-Ahsa, Saudi Arabia; 6 Department of Animal Diseases, Faculty of Veterinary Medicine, Aleppo University, Aleppo, Syria; 7 Division of Clinical Laboratory Diagnosis, Department of Animal Medicine, Faculty of Veterinary Medicine, Assiut University, Assiut, 71526 Egypt; 8 Department of Theriogenology, Obstetrics, and Artificial Insemination, Faculty of Veterinary Medicine, Aswan University, Aswan, 81528 Egypt

**Keywords:** Balanoposthitis, Clinical findings, Filariasis, Male camels, Testosterone, Sperm vitality and abnormalities

## Abstract

**Background:**

Camel filariasis induced variable clinical syndromes characterized by fever, lethargy, localized dermal lesions, loss of condition, and testicular and scrotal swelling. The objective of the present work focused on clarifying the diagnostic importance of clinical findings, serum testosterone, and semen analysis as well as blood smear and testicular histopathology as a differential tool between only balanoposthitis without filariasis male camels group (OnlyBp^gr^) and balanoposthitis-filariasis infected male camels group (BpFl^gr^). The study also monitored the associations between the severity of ticks’ infestations in investigated male camels and the occurrence of balanoposthitis only or balanoposthitis with filariasis.

**Results and conclusions:**

The study reported significant correlation between serum testosterone, serum cortisol, and sperm vitality and abnormalities percentages. The study included male camels (*n* = 250) classified into three groups: healthy control group (Cont^gr^; *n* = 30), OnlyBp^gr^ (*n* = 210), and BpFl^gr^ (*n* = 10). These male camels were clinically and laboratory examined, and skin scraping tests and testicular histopathology were conducted. The study confirmed the association of the changes in clinical findings, whole blood picture, serum testosterone, serum cortisol, and semen analysis, with OnlyBp^gr^ and BpFl^gr^. These changes were more prominent in BpFl^gr^ than in OnlyBp^gr^. Skin scraping test results revealed a higher severity of live ticks’ infestation in BpFl^gr^ than in OnlyBp^gr^ because, unlike OnlyBp^gr^, all camels in BpFl^gr^ (*n* = 10) were suffering from live ticks’ infestation. It also concluded the higher efficacy of histopathology of testicular tissues in male camels as a diagnostic tool for adult filaria in balanoposthitis-affected male camels than blood smear because all cases of camel filariasis in the current work were negative for microfilaria on microscopic examination of diurnal blood smear as well as testicular histopathology revealed detection of adult filaria in all camel filariasis associated with balanoposthitis. Strong correlation relationships were demonstrated between serum testosterone, serum cortisol, and semen analysis results. Positive correlations were reported between serum testosterone levels and sperm vitality percentages. However, negative correlations were stated between serum testosterone and each of serum cortisol and sperm abnormalities either in Cont^gr^, OnlyBp^gr,^ or BpFl^gr^.

## Background

In a variety of dry, semi-arid, and tropical regions in Asia, Africa, and Australia, dromedary one-humped camels (*Camelus dromedarius*) were well suited for severe environments [1–3]. These dromedary camels were valuable because they served a variety of functions, including providing food, milk, a means of transportation, and work [4].

Egypt did not give camels sufficient attention when it came to parasite infections, especially those caused by helminthes. However, according to Baraka et al. [5], helminthes were the main reason for reduced milk and meat production, declining male fertility, and declining female calving rates. Because camels were living in a difficult habitat, there had not been many studies on them. The herds’ non-sedentary lifestyle and continual movement in search of grass and water sources were to blame for this.

Products that are produced by camels, such milk, meat, and wool, might be impacted by a number of circumstances. Parasitic illnesses that had a negative impact on camels’ health were one of the main causes of the decrease in milk and meat production [6]. In camels, the heart, hepatic, pulmonary, and spermatic arteries, as well as the mesenteric lymph nodes and lymph vessels, were all affected by the filarial worm *Dipetalonema evansi* (*D. evansi*) [7, 8]. Different Aedes mosquito species could act as this parasite’s vectors. Mosquitoes consumed microfilariae while feeding on infected dromedary blood. The parasites then migrated to the mosquitoes’ chest muscles, where they continued to grow. After 10 days, presented larvae in mosquito’s proboscis had the ability to cause infection in a new host [9].

According to Hashem and Badawy [10], one of the most significant nematode-borne parasitic diseases that affected humans, animals, and birds was filariasis. The health, productivity, and working activity of camels were negatively impacted by hemoparasitic disorders such dipetalonemiasis [11].

One of the most significant diseases influencing camels in Upper Egypt was camel filariasis, which was caused by *D. evansi* and exhibited clinical symptoms that had an impact on camel reproductive function, working ability, and productivity [12]. Moreover, filaria infection in camels might be acute or chronic producing different clinical changes like loss of body weight, skin lesions, severe weakness, high body temperature and swelling of both scrotum and testis [12, 13].

Seasonal variations had a significant effect on the prevalence rate of *D. evansi* infection, with the summer months recording the greatest incidence and the winter months the lowest [14]. According to Borji et al. [15], the group of camels aged (4–5 years old) had the highest infection rate with *D. evansi*.

Reproductive efficacy was one of the key features associated with the raising of food animals. Traditional camel reproductive management involved having the owner’s camels handle mating throughout the rutting season. Due to their short mating season, difficult sperm collection, and later sexual maturation than other farm animals, dromedary camels really had poor reproductive performance [16].

Accordingly, the present work focused on clarifying the diagnostic importance of clinical findings, serum testosterone and semen analysis as well as blood smear and testicular histopathology as a differential tool between only balanoposthitis without filariasis male camels group (OnlyBp^gr^) and balanoposthitis-filariasis infected male camels group (BpFl^gr^). The study also monitored the associations between the severity of ticks’ infestations in investigated male camels and the occurrence of balanoposthitis only or balanoposthitis with filariasis. The study reported significant correlation between serum testosterone, serum cortisol, and sperm vitality and abnormalities percentages.

## Materials

### Animals

Two hundred and 50 mature male camels (*n* = 250) were involved through the current study They belonged to private farms in the Assiut and Aswan governorates, Egypt. The investigated male camels were taken kindly from the farm by permission that was taken from the farm owner. Their ages ranged between 6 and 10 years. Their body weight ranged from 650 to 740 kg. The study was carried out during the breeding season (December to March) during daytime. Camels were housed in an open yard. Animals were group-fed on a diet composed mainly of commercial concentrates mixture (12% crude protein and 70% Total Digestible Nutrients; TDN) (4 kg\head\day) in addition to roughage material of about 10 kg\head\day, which was Egyptian clover during the winter season (10 kg\head\day). Drinking water was offered all day. Based mainly on their histopathological findings as well as clinical findings and laboratory assays, the examined camels were classified into three main groups. Some of these camels were kept as a healthy control group (Cont^gr^; *n* = 30). The other camels were suffering from clinical signs of balanoposthitis (*n* = 220). Out of these balanoposthitis-infected camels (n = 220), 10 mature camels (4.55%) were diagnosed as positive for filariasis based mainly on their histopathological findings, and they were referred to as balanoposthitis-filariasis infected camels group (BpFl^gr^; *n* = 10). The other balanoposthitis-infected camels (*n* = 210) was referred to as only balanoposthitis without filariasis group (OnlyBp^gr^; n = 210). All camels were examined clinically, haematologically, and biochemically, and semen analysis was conducted. Regarding euthanasia methods, the male camels were slaughtered in a local abattoir at Aswan, Egypt. Histopathology of testicular tissues was carried out for 10 of the control group and for all balanoposthitis-affected camels (*n* = 220).

### Samples

Samples were obtained during the breeding season (December to March). The jugular vein was used to collect whole blood and serum samples, and all necessary steps were done during sample preparation and collection to ensure a precise evaluation of hematological and biochemical parameters. Serum samples were obtained and stored at − 20 °C for further hormonal analysis using test kits obtained from commercial sources [17].

Epididymal sperms were collected according to Shahin et al. [18] to assess sperm vitality and abnormalities.

Each selected animal was also examined for ectoparasites, mainly live ticks, by taking a skin scraping before and after treatment application [19].

### Clinical examination

The clinical examinations included mainly parameters of heart and respiratory rates, and rectal temperatures as well as rumen movements was done as described by Fowler [20]. According to Hutjens [21]; Hulsen [22]; Burfeind et al. [23]; Götze et al. [24], Khalphallah et al. [25, 26], and Elmeligy et al. [27], clinical scoring system and manure scoring of examined male camels were conducted. This monitoring included estimation of appetite score, rumen filling score (RFS), manure digestion score (MDS) and manure condition score (MCS). Feces were assessed for color, consistency, amount, fiber particle length, and shape.

### Complete blood picture indices

Complete blood pictures, including red blood corpuscles (RBCs), total leucocytic count (TLC), differential leukocytic count (DLC), hemoglobin (Hb), and packed cell volume (PCV) were manually estimated according to Coles [17]; Harvey [28]; and Latimer et al. [29].

### Microscopical examination of blood smears

According to Abdel-Rady [12]; Coles [17]; Weiss and Wardrop [30]; Zajac and Conboy [31], blood films of the wet, thin, and thick types, as well as concentration technique (Knott’s technique), were created for the diagnosis of microfilaria larvae (*D. evansi*).

#### Wet blood film

Two tiny droplets of blood were placed 1 centimeter apart on a clean, dry slide, covered with a cover slide, and examined at low power (× 10) to check for the mobility of microfilariae according to Abdel-Rady [12] and Coles [17].

#### Thin blood film

Two thin blood films were made, dried, fixed with absolute methanol for 5 minutes, dried again, and then stained for 30 minutes with Geimsa stain 10%. Excess stain was cleaned off, and a low-power lens examination was followed by a 100x oil immersion lens examination, according to Coles [17].

#### Thick blood film

Two drops of blood were placed on a clean, dry slide, speeded into a circle with a one-cm diameter, and then allowed to dry at room temperature. Dehemoglubinization was done by repeatedly submerging the slide in a container of distilled water, followed by 3–5 minutes of 100% methyl alcohol fixation and drying. 30 minutes of Geimsa dye 10% staining, and low power (10 x) and oil immersion lens (100 x) examination [12].

#### Concentration technique (Knott’s technique)

10 ml of 2% formalin and 1 ml of blood with EDTA were completely combined in a centrifuge tube. One drop of 0.1% methylene blue was added to the mixture and mixed before being transferred to a slide for microscopic examination. Thin and thick films were then prepared from the first sediment, fixed with absolute methyl alcohol, stained with Geimsa stain, and examined under a microscope. The mixture was centrifuged at 1000 rpm for 2 minutes [32].

#### Serum testosterone and cortisol hormone analysis

Through the use of commercial kits from Biodiagnostic, Cairo, Egypt, serum samples were examined using an enzyme-linked immunosorbent assay (ELISA-Sandwich Protocol) to determine the concentration of the hormone testosterone. Commercial radioimmunoassay kits from RandD Systems (MN 55413, Inc. at 614 McKinley Place NE in Minneapolis, Toll Free in the USA and Canada) were used to assess the levels of serum cortisol.

#### Sperm vitality and abnormalities

To assess sperm vitality, Moskovtsev and Librach [33] transferred a smear from the diluted semen samples to a glass slide and stained it with 5% eosin and 10% nigrosin stains. Two hundred spermatozoa from each sample were inspected under a light microscope, and the spermatozoa that were stained red were identified as being dead and counted. According to Menon et al. [34], the sperm morphological abnormalities included spermatozoa with aberrant or abnormal heads and tails.

#### Skin scraping test

Each male camel in Cont^gr^, OnlyBp^gr^, or BpFl^gr^ had a skin scraping taken in order to check for ectoparasites, primarily ticks. Each animal had a skin scraping taken in order to check for tick infestation. Then, samples prepared in 10% KOH solution were microscopically examined for adult ticks and identified according to Soulsby [35], Urquhart et al. [36], and Urquhart et al. [37]. In each case, the average number of ticks per microscopic field was estimated. The ticks’ infection was later determined by counting the live ticks on each cow and categorizing them as follows: + reflecting 1 to 10 live ticks, ++ reflecting 10 to 100 live ticks, and +++ reflecting more than 100 live ticks [13, 38–40].

#### Gross and histopathological examination

Male camels were slaughtered in a nearby abattoir in Aswan, Egypt. Testicular tissues were histopathologically examined for the 220 camels infected with balanoposthitis and the 10 members of the control group. In neighboring abattoir in Aswan, Egypt, the animals were slaughtered. There was a thorough inspection of the testicles. The testes were removed, cut into 1- to 2-cm-square pieces, and preserved in 10% neutral buffered formalin for later analysis. The samples were cleaned, dried in ethyl alcohol in increasing concentrations, cleaned in methyl benzoate, and then embedded in paraffin wax. Hematoxylin and eosin was used to stain a number of 3–5 μm thick paraffin sections, which were subsequently inspected [41].

#### Statistical analysis

SPSS statistical software program for Windows, version 10.0.1 (SPSS Inc., Chicago, IL., USA) was used for data analysis. The obtained data were described as mean ± SD. The data obtained from the clinical findings and laboratory analyses were analyzed using by general linear model repeated measures ANOVA and the significance level of results was set at *p* < 0.05. The significance of differences was evaluated between the means at Cont^gr^, OnlyBp^gr^ and BpFl^gr^. The correlation coefficient was calculated using Pearson Correlation at *p* < 0.05 or *p* < 0.01 between serum testosterone, serum cortisol, and percentages of sperm abnormalities and vitalities in examined male camels.

## Results

### Clinical findings

The control male camels showed normal clinical findings, as temperature, pulse and respiratory rates, rumen movements, appetite score, RFS, MDS and MCS were within the reference ranges. Significant elevations (*p* < 0.05) were observed in rectal temperature, pulse and respiratory rates, as well as a significant drop (p < 0.05) in rumen movements, appetite score, RFS, MDS and MCS was reported in each of OnlyBp^gr^ and BpFl^gr^ comparing with Cont^gr^. These significant changes were demonstrated between OnlyBp^gr^ and BpFl^gr^ for rectal temperatures, pulse and respiration rates, while they were absent for rumen movements, appetite score, RFS, MDS, and MCS. Values of rectal temperatures, pulse and respiration rates were remarkably (*p* < 0.05) elevated in BpFl^gr^ when they were compared with those in OnlyBp^gr^ (Table 1).

**Table 1:** Mean values (M ± SD) of temperature, pulse rates, respiration rates, rumen movements, appetite score, RFS, MCS, and MDS in Cont^gr^ (*n* = 30), OnlyBp^gr^ (*n* = 210), and BpFl^gr^ (*n* = 10) male camels

	Cont^gr^	OnlyBp^gr^	BpFl^gr^	Reference values
**Temperature** (°C)	37.37 ± 0.43^c^	38.78 ± 0.61^b^	40.16 ± 0.74^a^	(36–38)^20^ or (37.2 ± 0.77)^46^
**Pulse** (Beats/min)	33.18 ± 3.44^c^	40.62 ± 4.11^b^	44.77 ± 3.08^a^	(32–36)^20^ or (24–48/min)^43^
**Respiration** (Breaths/min)	13.33 ± 2.67^c^	24.88 ± 3.29^b^	33.07 ± 4.02^a^	(8–18)^44^ or (12.55 ± 0.30)^47^
**Rumen motility** (Movements/2 min)	3.35 ± 0.49^a^	1.44 ± 0.32^b^	1.36 ± 0.71^b^	(4.3 ± 0.14)^48^ or (3.65 ± 0.66)^49^
**Appetite score**	2.88 ± 0.23^a^	1.28 ± 0.37^b^	1.03 ± 0.56^b^	–
**RFS**	3.34 ± 0.26^a^	2.12 ± 0.27^b^	2.24 ± 0.41^b^	(1 = flat – 5 = distended or 3.0 ± 4.31 or ≤ 2/5)^45^
**MDS**	2.76 ± 0.31^a^	1.56 ± 0.25^b^	1.32 ± 0.32^b^	(2.5 to 3)^21^
**MCS**	2.91 ± 0.18^a^	2.36 ± 0.15^b^	2.16 ± 0.24^b^	(2.5 to 3)^21^

Cont^gr^: Control healthy group. OnlyBp^gr^: Only balanoposthitis without filariasis group. BpFl^gr^: Balanoposthitis-filariasis infected camels group. RFS: Rumen filling score. MDS: Manure digestion score. MCS: Manure condition score. ^a-c^Means within the same row with different superscript letters were significantly different (*P < 0.05*) in different male camels’ groups. Reference values according Fowler [20]; Hutjens [21]; Bhatt et al. [43]; Nielsen [44]; Bramley et al. [45]; Hamad et al. [46]; Hassan et al. [47]; Kamr et al. [48]; Mohamed et al. [49]

Out of 210 male camels in OnlyBp^gr^, most of the camels were suffering from loss of appetite (*n* = 180), fever (*n* = 185), polypnea (n = 185), tachycardia (n = 185), pale mucous membranes (*n* = 165), alopecia (*n* = 100), pruritis (n = 100) and emaciation (n = 100) (Table 2).

**Table 2:** The most common clinical findings in Cont^gr^ (*n* = 30), OnlyBp^gr^ (*n* = 210), and BpFl^gr^ (*n* = 10) male camels

	Cont^gr^	OnlyBp^gr^	BpFl^gr^
**Anorexia**	0 (0)^*^	180 (85.71)	10 (100)
**Fever (> 38 Ċ)**	0 (0)	185 (88.10)	8 (80)
**Tachy-cardia (> 36 b/m)**	0 (0)	185 (88.10)	8 (80)
**Poly-pnea (> 18 /m)**	0 (0)	185 (88.10)	0 (0)
**Abnormal lung sounds**	0 (0)	0 (0)	0 (0)
**Abnormal nasal discharges**	0 (0)	0 (0)	0 (0)
**Cough**	0 (0)	0 (0)	0 (0)
**Pale mucous membranes**	0 (0)	165 (78.56)	10 (100)
**Orchitis**	0 (0)	210 (100)	10 (100)
**Balanoposthitis**	0 (0)	210 (100)	10 (100)
**Corneal opacity**	0 (0)	0 (0)	0 (0)
**Abnormal lymph nodes**	0 (0)	0 (0)	0 (0)
**Melena**	0 (0)	0 (0)	0 (0)
**Diarrhea**	0 (0)	0 (0)	0 (0)
**Alopecia**	0 (0)	100 (47.71)	10 (100)
**Pruritis**	0 (0)	100 (47.71)	10 (100)
**Emaciation**	0 (0)	100 (47.71)	2 (20)

Cont^gr^: Control healthy group. OnlyBp^gr^: Only balanoposthitis without filariasis group. BpFl^gr^: Balanoposthitis-filariasis infected camels group. ^*^Number of investigated male camels (%)

**Fig. 1:**
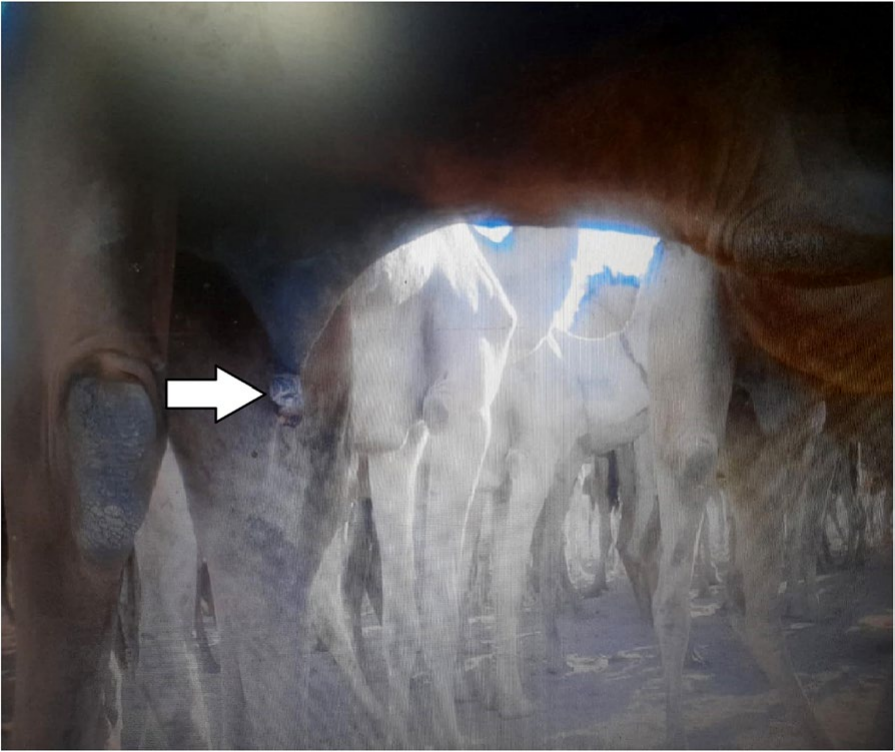
Balanoposthitis-filariasis-infected male camels showed balanoposthitis (White arrow)

Out of 10 male camels in BpFl^gr^, anorexia, pale mucous membranes, alopecia, and pruritis were observed in all filariasis-positive camels. Fever, polypnea and tachycardia were described in most of the male camels with the acute form of filariasis (*n* = 8). Emaciations were observed in chronic cases of camel filariasis (*n* = 2) (Table 2; Fig. 1).

All male camels either in OnlyBp^gr^ or BpFl^gr^ had normal lymph nodes and normal lung sounds as well as cough, abnormal nasal discharges, corneal opacity, melena and diarrhea were not described. In contrast, All OnlyBp^gr^ and BpFl^gr^ had signs of orchitis and balanoposthitis (Table 2).

### Complete blood picture indices

Whole blood picture indices i.e. RBCs, Hb, PCV, TLC, and DLC, were within the reference ranges in control healthy male camels. OnlyBp^gr^ had normal values of RBCs, Hb, and PCV, while DLC showed neutrophilic leukocytosis. BpFl^gr^ had lower values of RBCs, Hb and PCV as well as eosinophilic leukocytosis was also reported. RBCs, Hb, and PCV values were remarkably (*p* < 0.05) dropped in BpFl^gr^ compared to Cont^gr^ and OnlyBp^gr^. TLC was significantly (p < 0.05) increased in OnlyBp^gr^ and BpFl^gr^ when compared with control healthy camels. However, these significant changes were not reported between OnlyBp^gr^ and BpFl^gr^. Furthermore, no significant alterations were demonstrated between Cont^gr^ and OnlyBp^gr^ for values of RBCs, Hb, and PCV (Table 3).

**Table 3:** Mean values (M ± SD) of whole blood picture indices in Cont^gr^ (*n* = 30), OnlyBp^gr^ (*n* = 210) and BpFl^gr^ (*n* = 10) male camels

	Cont^gr^	OnlyBp^gr^	BpFl^gr^	Reference values
**RBCs** (_X_10^12^/L)	11.34 ± 3.63^a^	10.51 ± 2.63^a^	6.61 ± 1.34^b^	(7.5–12)^20^ or (15.05 ± 2.10)^55^
**Hb** (g/L)	136.08 ± 3.48^a^	121.28 ± 4.11^a^	82.28 ± 4.11^b^	(120–150)^20^
**PCV** (L/L)	0.31 ± 0.03^a^	0.34 ± 0.05^a^	0.32 ± 0.02^a^	(0.26–0.38)^20^
**TLC** (_X_10^9^/L)	11.44 ± 2.26^b^	17.69 ± 3.96^a^	19.33 ± 4.81^a^	(6–13.5)^20^ or (11.12 ± 1.89)^58^
**Neutrophils (%)**	56.05 ± 3.11^b^	65.36 ± 4.08^a^	55.73 ± 4.66^b^	(50–60)^20^ or (52.25 ± 0.85)^57^
**Lymphocytes (%)**	35.87 ± 4.82^a^	31.51 ± 3.88^a^	32.45 ± 2.87^a^	(30–45)^20^ or (34.25 ± 1.37)^57^
**Monocytes (%)**	5.26 ± 1.12^a^	3.5 ± 1.68^a^	4.76 ± 1.03^a^	(2–8)^20^ or (6.5 ± 0.86)^57^
**Eosinophil (%)**	2.32 ± 0.45^b^	2.25 ± 1.82^b^	12.51 ± 3.29^a^	(5.31 ± 0.1)^55^ or (6.5 ± 0.64)^57^
**Band cells (%)**	0.51 ± 0.46^a^	0.58 ± 0.33^a^	0.61 ± 0.29^a^	(2.53 ± 0.11)^55^

Cont^gr^: Control healthy group. OnlyBp^gr^: Only balanoposthitis without filariasis group. BpFl^gr^: Balanoposthitis-filariasis infected camels group. RBCs: Red blood corpuscles. Hb: Haemoglobin. PCV: Packed cell volume. TLC: Total leucocytic count. ^ab^Means within the same row with different superscript letters were significantly different (*P < 0.05*) in different male camels’ groups. Reference values according to Fowler [20]; Adah et al. [55]; Poonia et al. [57]; Khalphallah et al. [58]

Microscopic examination of blood samples in diurnal using different methods of blood film preparation i.e., thin film, thick film, and concentration technique, revealed the percentage of infection (Overall prevalence) with microfilaria was 0% in all examined male camels, including Cont^gr^, BpFl^gr^ comparing with and OnlyBp^gr^ (Table 4).

**Table 4:** Overall prevalence (%) of Camels filariasis (*Dipetalonema evansi*) in Cont^gr^ (*n* = 30), OnlyBp^gr^ (*n* = 210) and BpFl^gr^ (*n* = 10) male camels based on blood films data

Male camels groups	Number of Examined male camels	Number of positive	Prevalence (%)
**Cont**^**gr**^	30	0	0
**OnlyBp**^**gr**^	210	0	0
**BpFl**^**gr**^	10	0	0

Cont^gr^: Control healthy group. OnlyBp^gr^: Only balanoposthitis without filariasis group. BpFl^gr^: Balanoposthitis-filariasis infected camels group

### Serum testosterone and cortisol hormones

OnlyBp^gr^ and BpFl^gr^ had significantly (*p* < 0.05) lower serum testosterone values than those in Cont^gr^. These significant changes were absent between OnlyBp^gr^ and BpFl^gr^. These values were lower than their reference ranges. Serum levels of cortisol were significantly (*p* < 0.05) elevated in OnlyBp^gr^ and BpFl^gr^ when compared with their values in Cont^gr^. These significant differences were not observed between OnlyBp^gr^ and BpFl^gr^ whereas their serum cortisol values were higher than their reference ranges (Table 5).

**Table 5:** Mean values (M ± SD) of serum testosterone and cortisol hormones in Cont^gr^ (*n* = 30), OnlyBp^gr^ (*n* = 210) and BpFl^gr^ (*n* = 10) male camels

	Cont^gr^	OnlyBp^gr^	BpFl^gr^	Reference values
**Testosterone** (ng/ml)	4.14 ± 0.26^a^	2.80 ± 0.49^b^	2.78 ± 0.45^b^	(2.8–24)^60^ or (10–15)^61^
**Cortisol** (nmol/L)	35.97 ± 4.55^b^	49.27 ± 6.28^b^	68.79 ± 7.41^a^	(38.17 ± 3.99)^68^

Cont^gr^: Control healthy group. OnlyBp^gr^: Only balanoposthitis without filariasis group. BpFl^gr^: Balanoposthitis-filariasis infected camels group. ^ab^Means within the same row with different superscript letters were significantly different (*P < 0.05*) in different male camels’ groups. Reference values according to Tibary and Anouassi [60]; Deen [61]; Saeb et al. [68]

### Sperm vitality and abnormalities

The percentages of vital sperms were significantly (p < 0.05) higher, however, the percentages of sperms abnormalities were significantly (p < 0.05) lower in Cont^gr^ their values in OnlyBp^gr^ and BpFl^gr^. No remarkable changes were reported between OnlyBp^gr^ and BpFl^gr^ either for sperms vitality percentages or for sperm abnormalities percentages (Table 6).

**Table 6:** Mean values (M ± SD) of either normal or abnormal sperm percentages in Cont^gr^ (*n* = 30), OnlyBp^gr^ (*n* = 210) and BpFl^gr^ (*n* = 10) male camels

	Cont^gr^	OnlyBp^gr^	BpFl^gr^
**Sperm vitality**	58.45 ± 5.11^a^	40.70 ± 5.14^b^	38.82 ± 4.38^b^
**Sperm abnormalities**	17.23 ± 2.4^b^	25.85 ± 3.76^a^	27.55 ± 4.13^a^

Cont^gr^: Control healthy group. OnlyBp^gr^: Only balanoposthitis without filariasis group. BpFl^gr^: Balanoposthitis-filariasis infected camels group. ^ab^Means within the same row with different superscript letters were significantly different (*P < 0.05*) in different male camels’ groups

### Skin scraping test

Skin scraping test results revealed that Cont^gr^ was free from ticks under the microscope (−). The severity of ticks’ infestation was more clear in OnlyBp^gr^ and BpFl^gr^. Out of 210 camels in OnlyBp^gr^, 100 male camels had ticks, whereas 10–100 (++) adult ticks were detected microscopically. All camels in BpFl^gr^ (*n* = 10) were suffering from live ticks’ infestation whereas more than 100 (+++) adult ticks were detected microscopically. The number of live ticks detected microscopically was significantly (*p* < 0.05) higher in OnlyBp^gr^ and BpFl^gr^ when they compared with their values in Cont^gr^. The number of live ticks was remarkably elevated in BpFl^gr^ compared to OnlyBp^gr^ (Table 7)_._

**Table 7:** Skin scraping test results and severity of ticks’ infestation in Cont^gr^ (*n* = 30), OnlyBp^gr^ (*n* = 210) and BpFl^gr^ (*n* = 10) male camels

	Cont^gr^	OnlyBp^gr^	BpFl^gr^	Reference values
**Ticks infestation**	**-** (0)^c^	++ (87 ± 13)^b^	+++ (188 ± 42)^a^	(−: No live ticks, +: 1–10 live ticks, ++: 10–100 live ticks, +++: More than 100 live ticks)^38,39^

Cont^gr^: Control healthy group. OnlyBp^gr^: Only balanoposthitis without filariasis group. BpFl^gr^: Balanoposthitis-filariasis infected camels group. ^abc^Means within the same row with different superscript letters were significantly different (*P < 0.05*) in different male camels’ groups. Reference values according to Liebish et al. [38]; Greiner [39]

### Gross and histopathological examination

In the present study, out of the infected camels with balanoposthitis (*n* = 220), 10 camels (4.55%) had lesions and white, slender shape of *D. evansi* as well as most infections with these mature nematodes were seen in the testes of male camels with balanoposthitis.

**Fig. 2:**
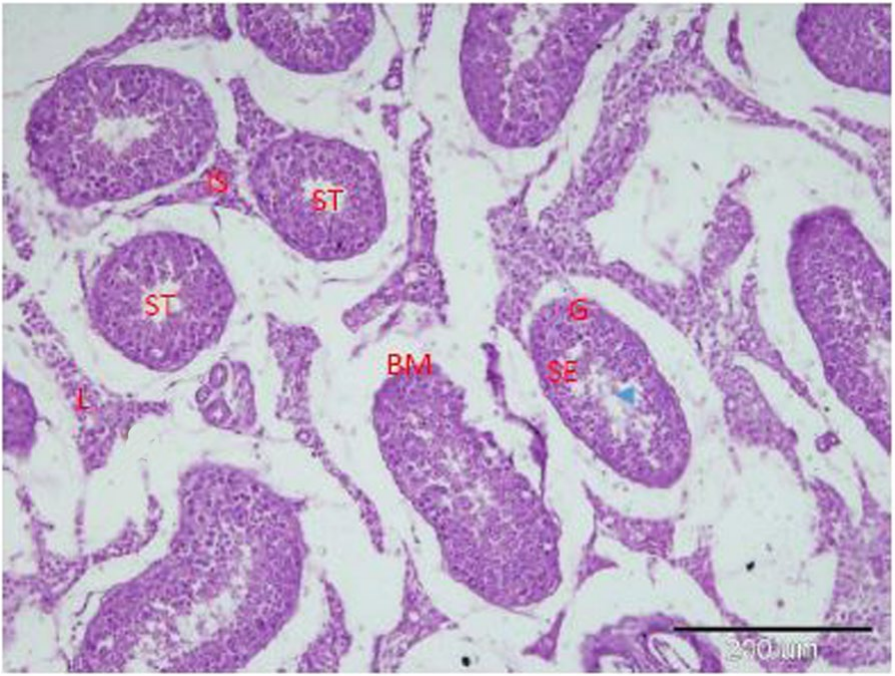
A photomicrograph of a section in the camel testis showed normal morphological structures of seminiferous tubules (ST) lined by germinal epithelium (G) and Sertoli cells (SE) resting on the basement membrane (BM). Sperms (arrowhead) were seen in the lumen and interstitial spaces (IS) in between the tubules, which contained interstitial cells of Leydig (L), blood vessels, and lymph vessels

**Fig. 3:**
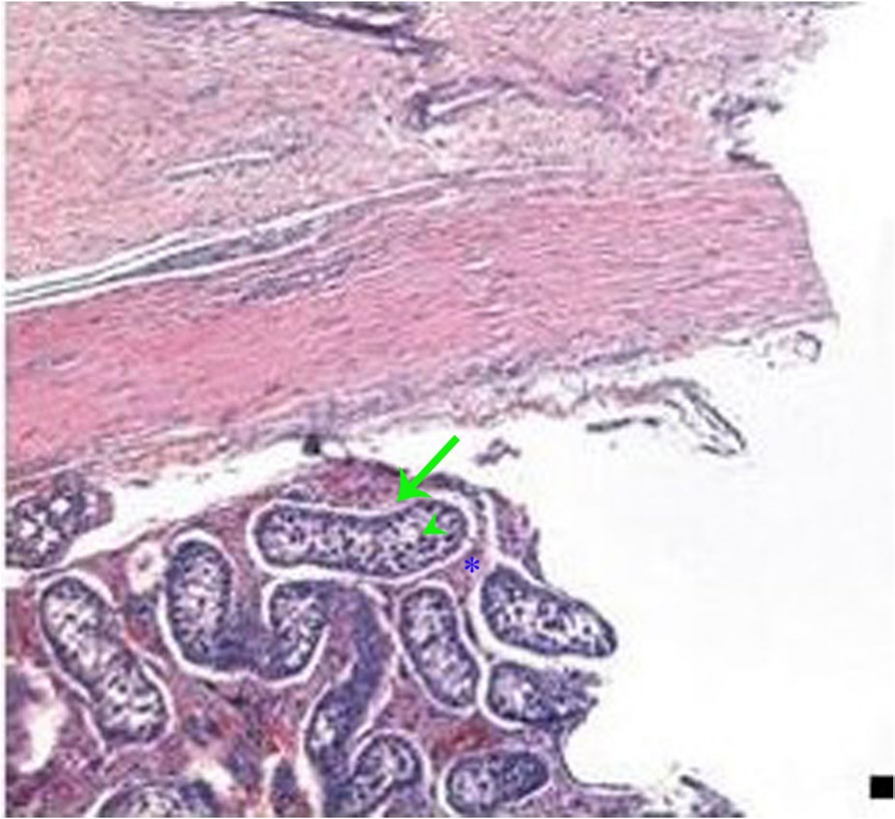
A photomicrograph of a section in camel testis, which had balanoposthitis without filarial infection, showing necrosis and atrophy of seminiferous tubule (arrow). There were incomplete spermatogenesis (arrowhead) and interstitial mononuclear inflammatory cells infiltration (star)

**Fig. 4:**
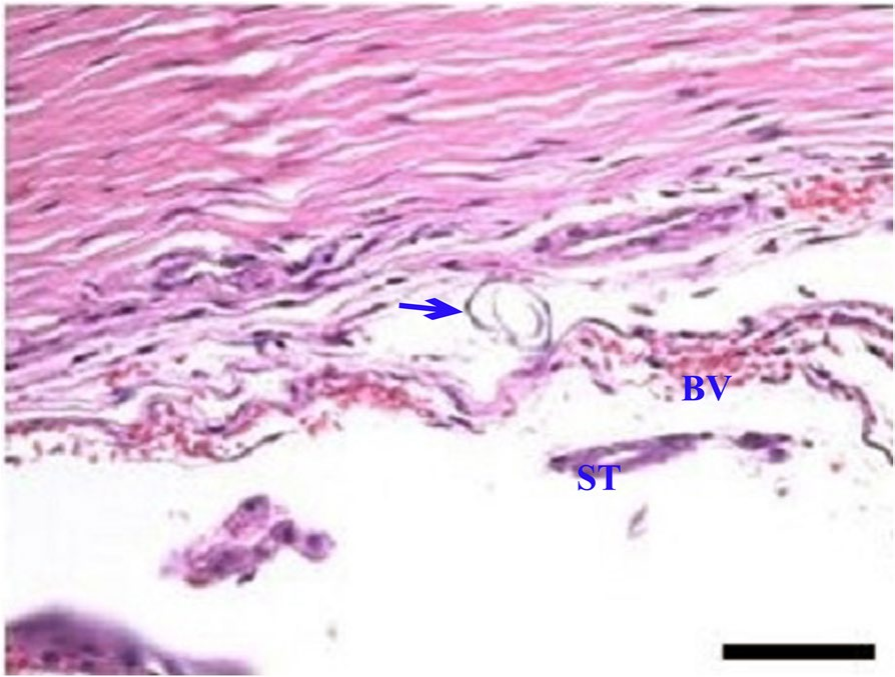
A photomicrograph of a section in camel testis, which had balanoposthitis, showing a section of *Dipetalonema evansi* (arrow) is seen free in the luminal area of an artery in the testicular parenchyma. Marked reduction of the number of seminiferous tubules (ST); shedding and degenerating cells had dark stained nuclei. There was a wide interstitial tissue gap, congested blood vessels (BV), and interstitial edema associated with inflammatory cells infiltration

Histopathology of the testicular tissues of the healthy male camels showed normal morphological structures, seminiferous tubules lined by germinal epithelium, and Sertoli cells resting on the basement membrane. Sperms were seen in the lumen and interstitial spaces in between the tubules, which contained interstitial cells of Leydig, blood vessels, and lymph vessels (Fig. 2). On the other hand, histopathology of the testicular tissues of the balanoposthitis-affected male camels without filarial infection in OnlyBp^gr^ showed necrosis and atrophy of seminiferous tubule. Furthermore, incomplete spermatogenesis and interstitial mononuclear inflammatory cells infiltration were observed (Fig. 3). In contrast, histopathology of the testicular tissues of the infected male camels with balanoposthitis and filariasis in BpFl^gr^ showed the presence of mature nematodes of filaria in the testes of male camels with balanoposthitis. It described a section of *Dipetalonema evansi* (is seen free in the luminal area of an artery in the testicular parenchyma. Marked reduction of the number of seminiferous tubules and shedding and dark stained nuclei of degenerating cells were seen. A wide interstitial tissue gap, congested blood vessels, and interstitial edema were associated with inflammatory cell infiltration (Fig. 4).

### Correlations between serum testosterone, serum cortisol, percentages of sperm abnormalities, and vitalities in examined male camels

Significant correlations were demonstrated between serum testosterone, serum cortisol, sperm vitality, and sperm abnormalities (Table 8)_._

**Table 8:** Pearson correlation coefficient between serum testosterone, serum cortisol, percentages of sperm abnormalities and vitalities in examined male camels

	Sr. testosterone	Sr. Cortisol	Spr. Vit. (%)	Spr. Abnr. (%)
**Sr. testosterone**		*r* = − 0.62^**^	*r* = 0.75^**^	*r* = − 0.66^**^
		*P*_*v*_ = 3.21 _X_ 10^−5^	*P*_*v*_ = 5.30 _X_ 10^−8^	*P*_*v*_ = 6.98 _X_ 10^−6^
**Sr. Cortisol**			*r* = − 0.63^**^	*r* = 0.56^**^
			*P*_*v*_ = 2.49 _X_ 10^−5^	*P*_*v*_ = 2.45 _X_ 10^−4^
**Spr. Vit. (%)**				*r* = − 0.71^**^
				*P*_*v*_ = 7.25 _X_ 10^−7^
**Spr. Abnr. (%)**				

Sr. Serum. Spr. Vit. (%): Sperm vitality Spr. Abnr. (%): Sperm abnormalities percentages. P_v_: *P* value. ^**^Significant (two-tailed) *p* < 0.01. Gray backgrounds referred to correlation between the same parameter e.g. Sr. testosterone and Sr. testosterone where there was no correlation. Diagonal backgrounds referred that this correlation was previously reported in the previous row e.g. correlation between Sr. testosterone and Sr. Cortisol was reported in row 1 and so there was no need to repeat it at row 2

Positive correlations were reported between serum testosterone levels and sperm vitality percentages, however, negative correlations were stated between serum testosterone and each of serum cortisol and sperm abnormalities either in Cont^gr^, OnlyBp^gr^ or BpFl^gr^. A significant elevation in serum testosterone in Cont^gr^ was associated with a significant raise in sperm vitality percentages. Furthermore, in OnlyBp^gr^ and BpFl^gr^, significant drop in serum testosterone values was associated with a significant drop in sperm vitality percentages. A significant elevation in serum testosterone in Cont^gr^ was associated with a significant drop in each of serum cortisol and sperm abnormalities percentages. A significant drop in serum testosterone values was associated with a significant elevation in sperm vitality percentages in OnlyBp^gr^ and BpFl^gr^ (Table 8)_._

Serum cortisol concentrations were positively correlated with sperm abnormalities percentages. However, they were negatively correlated with sperm vitality percentages in Cont^gr^, OnlyBp^gr,^ or BpFl^gr^. A remarkable elevation in serum cortisol was associated with a remarkable increase in sperm abnormalities in OnlyBp^gr^ and BpFl^gr,^ while a significant drop in serum cortisol was associated with a significant drop in sperm abnormalities percentages in Cont^gr^. A remarkable increase in serum cortisol was associated with a remarkable drop in sperm vitality percentages in OnlyBp^gr^ and BpFl^gr,^ while a significant drop in serum cortisol was associated with a significant increase in sperm vitality percentages in Cont^gr^ (Table 8)_._

Negative correlations were observed between sperm vitality percentages and sperm abnormalities percentages either in Cont^gr^, OnlyBp^gr^ or BpFl^gr^. The significant reduction in sperm abnormalities percentages was associated with a significant elevation in sperm vitality percentages in Cont^gr^. A remarkable elevation in sperm abnormalities percentages was associated with a remarkable reduction in sperm vitality percentages in OnlyBp^gr^ and BpFl^gr^ (Table 8)_._

## Discussion

### Clinical findings

Several clinical manifestations correlated with camel filariasis include scrotal and testicular enlargement, localized skin lesions, severe exhaustion, emaciation, and high body temperatures [13, 42]. Referring to the current study, the control male camels showed normal clinical findings whereas temperature, pulse and respiratory rates, rumen movements, appetite score, RFS, MDS, and MCS were within the reference ranges mentioned by Fowler et al. [20]; Hutjens [21]; Bhatt et al. [43]; Nielsen [44]; Bramley et al. [45]; Hamad et al. [46]; Hassan et al. [47]; Kamr et al. [48]; Mohamed et al. [49]. On the other side, OnlyBp^gr^ and BpFl^gr^ had significant elevations in rectal temperature, pulse and respiratory rates as well as a significant drop in rumen movements, appetite score, RFS, MDS and MCS comparing with Cont^gr^. These significant changes were demonstrated between OnlyBp^gr^ and BpFlgr for rectal temperatures, pulse, and respiration rates, while they were absent for rumen movements, appetite score, RFS, MDS and MCS. Rectal temperatures, pulse, and respiration rates were remarkably elevated in BpFlgr when they compared with those in OnlyBpgr. These results were confirmed by Abdel-Rady [12]; Muhammad et al. [50]; Kachhawaha et al. [51]. Muhammad et al. [50]; Kachhawaha et al. [50] hypothesized that the increase in body temperature might be caused by the stress because of microfilaria migration in the host’s body. An increase in heart rate and respiratory rate was used to compensate up for anemia and meet the body’s oxygen needs.

The previous reports mentioned that camels naturally infected with microfilaria displayed fever, rigidity in their movements, emaciation, pale mucous membranes, and decreased appetite [51, 52]. These findings and results were mentioned also by Abdel-Rady [12]; Karram et al. [13]; Agag et al. [52] and supporting the current results, which referred to variations in percentages of involved male camels that had clinical abnormalities either in OnlyBp^gr^ and BpFl^gr^. Most of the camels in OnlyBp^gr^ suffered from loss of appetite, fever, polypnea, tachycardia, pale mucous membranes, alopecia, pruritis and emaciation. On the other hand, all infected male camels in BpFl^gr^ had anorexia, pale mucous membranes, alopecia, and pruritis. BpFl^gr^ had signs of Fever, polypnea, and tachycardia that were described in most male camels with acute filariasis. Emaciations were observed in chronic cases of camel filariasis. Moreover, all male camels either in OnlyBp^gr^ or BpFl^gr^ had normal lymph nodes and normal lung sounds as well as cough, abnormal nasal discharges, corneal opacity, melena and diarrhea were not described. All OnlyBp^gr^ and BpFl^gr^ had signs of orchitis and balanoposthitis. In contrast, Muhammad et al. [50]; Kachhawaha et al. [51] reported that clinical examination in camel filariasis recorded swollen pharyngeal lymph nodes. Abdel-Rady [12] differentiated between the acute and chronic forms of camel filariasis, with the chronic form characterized by generalized debility and emaciation in camels. The acute form is distinguished by unwillingness to move, severe orchitis, and balanoposthitis. Some similarities in clinical finding between the current study and what were reported by Abdel-Rady [12] might be attributed to the fact that the organs i.e. mainly testis and prepuce, affected by the parasites in the current study were the same organs affected in camels studied by these investigators. Furthermore, Chhabra and Gupta [53] added that mild infections of *D. evansi* were not diagnosed. Cachexia, possibly orchitis, neurological manifestations, and probably death were the signs of severe illnesses. Dipetalonemiasis symptoms included reduced appetite, fatigue, pale mucous membranes, orchitis, arteriosclerosis, spermatic cord aneurysms, and heart failure.

### Blood picture indices

The number of positive filariasis blood films at midnight was much higher than what was collected at midday [54, 55]. The prevalence of *D. evansi* microfilariae in blood samples ranged from 0.88 to 46.7%, according to other earlier investigations [56]. The control camels in the current study had normal values of blood pictures indices that were supported by Fowler [20]; Adah et al. [55]; Poonia et al. [57]; Khalphallah et al. [58]. OnlyBp^gr^ in the current work had normal values of RBCs, Hb and PCV while DLC showed neutrophilic leukocytosis. BpFl^gr^ was suffering from anemia as they had lower values of RBCs, Hb and PCV as well as eosinophilic leukocytosis was also reported. Comparing different investigated male camel groups, BpFl^gr^ had lower values of RBCs, Hb and PCV comparing with Cont^gr^ and OnlyBp^gr^. TLC was significantly increased in OnlyBp^gr^ and BpFl^gr^ compared to control healthy camels. Hence, these significant changes were absent between OnlyBp^gr^ and BpFl^gr^.

Furthermore, Cont^gr^ and OnlyBp^gr^ had no significant difference between each other either for values of RBCs, Hb or PCV. These results were supported by Muhammad et al. [50]; Kachhawaha et al. [51]. On the other hand, the severity of the hemoglobin decrease caused by microfilariae feeding on the blood in peripheral blood was dependent on the number of worms present. The most significant hematological change was eosinophilia [50].

Microscopic examination of blood samples in diurnal i.e., at mid-day, using different methods of blood film preparation, revealed that the percentage of microfilaria infection was 0% in all examined male camels i.e., Cont^gr^, OnlyBp^gr^, and BpFl^gr^. These results were supported by Adah et al. [55], who revealed a correlation between the number of microscopically positive cases for camel filariasis and the number of blood samples collected during the day and night. Additionally, the blood samples collected at night gave more positive cases although the number of blood samples collected during the day was higher than that of night. This may be due to the nocturnal periodicity of *D. evansi* microfilariae, as 8 of 143 examined blood samples (5.59%) taken at night were positive. This means that, compared to the diurnal sample, nocturnal sampling increased the likelihood of finding microfilaria. Saleh [54] also added that microfilariae were 10 times more likely to be found in blood samples taken at night than in those taken during the day. El-Amin et al. [59] indicated that microfilariae had a biphasic periodicity pattern with peak concentrations around 8 p.m. and between 4 and 6 a.m. These findings were different from those noted by Karram et al. [13], who showed that the microfilairae count in camel blood was unaffected by time of day or night but was influenced by a febrile state. This nocturnal behavior of microfilariae might be owed to chemotactic substances found in *D. evansi* larvae that are altered by day and night.

### Serum testosterone and cortisol hormones

All diseased camels through the present work suffered from orchitis and balanoposthitis that reflected serum levels of testosterone and cortisol. OnlyBp^gr^ and BpFl^gr^ had significantly lower values of serum testosterone than those in Cont^gr^. These significant changes were absent between OnlyBp^gr^ and BpFl^gr^. These values were lower than their reference ranges mentioned by Tibary and Anouassi [60]; Deen [61]. Compared to the non-breeding season, testosterone levels in blood plasma and testicular tissue increased dramatically during the breeding season. Testicular tissue also had a greater testosterone concentration than blood [62]. Males’ testosterone levels are frequently necessary for proper spermatogenesis [63, 64] and reproductive tract function [65].

Additionally, testosterone was crucial for avoiding apoptotic cell death in tissues that depended on androgens [66, 67]. Both the proliferation and the level of testosterone in the testis were negatively correlated with the degree of apoptosis. As a result, testosterone played a crucial role as both a testicular product and a regulator of the testis’s functions [64]. Regarding to the current study, serum levels of cortisol were significantly elevated in OnlyBp^gr^ and BpFl^gr^ when compared with their values in Cont^gr^. These significant differences were not observed between OnlyBp^gr^ and BpFl^gr,^ whereas their serum cortisol values were higher than their reference ranges mentioned by Saeb et al. [68]. Since excess cortisol was synthesized and released into the systemic circulation under stressful conditions, measuring blood cortisol concentration was utilized as a standard approach for detecting stress in farm animals [69]. In general, cortisol supports energy consumption, reproduction, immunological response, inflammatory processes, growth, and brain function to assist the body `in maintaining homeostasis. Nevertheless, sustained elevations in glucocorticoid levels had a detrimental effect on immunological response or reproductive activity [70, 71].

### Sperm vitality and abnormalities

Orchitis and balanoposthitis were described in all diseased camels in both OnlyBp^gr^ and BpFl^gr^, as the previous reports mentioned that **t**he testes were in charge of spermatozoa production and androgen secretion. Cellular differentiation took place over a long time to produce the spermatozoon. Based on biochemical and cytochemical changes, morphofunctional modifications arose during this process [72, 73]. Testosterone in males is necessary for optimal spermatogenesis [63, 64] and proper reproductive tract function [65]. The present study reported a significant reduction in sperm vitality percentages as well as a significant increase in sperm abnormalities percentages in OnlyBp^gr^ and BpFl^gr^ compared to Cont^gr^. No remarkable changes were reported between OnlyBp^gr^ and BpFl^gr^ either for sperm vitality percentages or for sperm abnormalities. These results were supported by previous research by Suresh et al. [74], in which androgen deprivation caused an immediate arrest in the meiotic transition of primary spermatocytes to spermatids, thereby halting sperm production. According to a number of studies, testosterone also affects the epididymis’ size and function, impacting the maturation and survival of spermatozoa during epididymal transit [75, 76].

Additionally, the production of certain caput and cauda epididymal proteins was impacted by the testosterone hormone. Some of these proteins may be crucial for spermatozoa’s development, storage, and acquisition of fertilization potential [77]. On the other hand, in such a natural mating reproductive management, poor male fertility could result in significant pregnancy failures. One of the main causes of slowing down and disrupting the spermatogenesis processes, which led to low sperm concentration and quality, was testicular degeneration, which might be brought on by infections, chemicals, and environmental factors. It was demonstrated that *D. evansi*-caused filariasis could result in orchitis and spermatic cord hematomas [78, 79].

### Skin scraping test

Camels with both the acute and chronic forms of camel filariasis have hard ticks on them. Hard tick infestations were found in 11 out of 13 positive cases; no lakes or mosquito populations were found in surrounding areas of the positive cases [12]. This observation agreed with the results obtained by Ramadan [80], who stated that Hard ticks collected from microfilaria-infected camels had the microfilariae separated from their mouth and body cavities. These results confirmed the findings of the current study in which skin scraping test results revealed a higher severity of live ticks’ infestation in BpFl^gr^ than that in OnlyBp^gr^ because, unlike OnlyBp^gr^, all camels in BpFl^gr^ were suffering from live ticks’ infestation. According to Liebish [38]; Greiner [39]; Bowman [40], Cont^gr^ were free from ticks under the microscope (−). The severity of ticks’ infestation was clearer in OnlyBp^gr^ (++) and BpFl^gr^ (+++). The numbers of live ticks detected microscopically were significantly higher in BpFl^gr^ when comparedto their values in OnlyBp^gr^_._ On the other hand, Abdel-Rady [12] confirmed the high incidence rates of filariasis in the El-Wady El-Gaded governorate, which might be attributed to the presence of hard ticks, the disease’s primary vector, which was present on all suspected and infected camels in a heavy manner (microscopically confirmed and clinically ill), as well as other predisposing factors like breeding, fluctuation of temperature in day and night, and management systems, were mentioned.

### Gross and histopathological examination

It was stated that *D. evansi* filariasis could produce orchitis and spermatic cord hematomas by compromising spermatogenesis processes, resulting in low sperm concentration and quality [78, 79]. Moreover, in the arteries of the spermatic cords, white adult nematodes were discovered during macroscopic examinations of infected testis [81]. In the present study, out of the infected camels with balanoposthitis (*n* = 220), 10 camels (BpFl^gr^; 4.55%) had lesions and white, slender shape of *D. evansi* as well as most of the infections with these mature nematodes were seen in the testes of male camels with balanoposthitis (BpFl^gr^). Kheirandish et al. [81] supported the current results, whereas they mentioned that the five infected samples in male camels had gross lesions and white, slender *D. evansi.* Sazmand et al. [82] reported that 13.89% of camels contained adult nematodes in one organ. They found that mature *D. evansi* nematode infections tended to occur more frequently in the testes and that males were considerably more susceptible than females to have macroscopic adult worm infections.

On the other hand, *D. evansi* filariasis could additionally affect other tissues such as the mesentery, lymph nodes, right auricle, and pulmonary arteries [83]. The current study reported the presence of mature adult filaria in the camels’ testes in BpFl^gr^. In contrast, the presence of mature nematode in the testicular tissue was a rare condition. Another helminth that could infect the testis was *Dirofilaria*. In other research, *D. immitis* and *D. repens* were found in the spermatic cord, epididymis, and scrotum [84–86]. However, *D. repens* frequently caused subcutaneous nodules, while *D. immitis* mostly affected the cardiovascular system. Only human, cat, and dog infections with *Dirofilaria* were reported [87]. On the other hand, testicular dirofilariasis was a rare illness, and most reported cases involved the subcutaneous form in the scrotum. However, according to one study [88], *D. evansi* was present in the testicular tissue of 50% of the rats. Accordingly, the filariasis produced by *D. evansi* represented a unique parasitic manifestation of adult nematodes in the tissues of the male camels’ testicular organs.

Regarding the current study, histopathology of the testicular tissues of the healthy male camels showed normal morphological structures of the seminiferous tubules. Sperms were seen in the lumen and interstitial spaces between the tubules, which contained interstitial cells of Leydig, blood vessels, and lymph vessels. On the other hand, in OnlyBp^gr^, histopathology of the testicular tissues of the balanoposthitis-affected male camels without filarial infection showed necrosis and atrophy of seminiferous tubule. Furthermore, incomplete spermatogenesis and interstitial mononuclear inflammatory cell infiltration were observed. In contrast, the infected male camels in BpFl^gr^ showed histopathologically the presence of mature nematodes of filaria in the testes of male camels with balanoposthitis. Marked reduction of the number of seminiferous tubules as well as shedding and degenerating cells with dark stained nuclei were seen.

Moreover, a wide interstitial tissue gap, congested blood vessels, and interstitial edema associated with inflammatory cells infiltration were reported. These results were confirmed by Chhabra and Gupta [53]; Kheirandish et al. [81]; Sazmand et al. [82]. According to Kheirandish et al. [81], spermatogenetic activity, increased interstitial space tubules, obstruction of testicular blood vessels by parasites, hypertrophy of blood vessels, degenerative and necrosis changes in the tubules, and destruction of the spermatogenic cells were all noted through their histopathological examination of *D. evansi*-infected testis. They also added that all stages of spermatogenic cells occurred in most seminiferous tubules, such as spermatogonia, primary and secondary spermatocytes, and round spermatids. In most of the seminiferous tubules, spermatozoa were observed in their lumens. Degenerative alterations in the seminiferous tubules had been observed along with the presence of adult nematodes in the spermatic cord. Moreover, Sazmand et al. [82] showed fibrosis, atrophy, and inflammation in testes from infected camels with *D. evansi*. Additionally, they noted the necrosis and sloughing of germ cells, arterial wall inflammation with hemorrhage in a few interstitial tissue locations, and eosinophil and lymphocyte infiltration.

### Correlations between serum testosterone, serum cortisol, percentages of sperm abnormalities, and vitalities in examined male camels

Significant correlations were demonstrated between serum testosterone, serum cortisol, sperm vitality, and sperm abnormalities_._ Positive correlations were reported between serum testosterone levels and sperm vitality percentages. However, negative correlations were stated between serum testosterone and each of serum cortisol and sperm abnormalities either in Cont^gr^, OnlyBp^gr,^ or BpFl^gr^. Serum cortisol concentrations were positively correlated with sperm abnormalities percentages. However, they were negatively correlated with sperm vitality percentages in Cont^gr^, OnlyBp^gr,^ or BpFl^gr^. Negative correlations were observed between sperm vitality percentages and sperm abnormalities percentages either in Cont^gr^, OnlyBp^gr,^ or BpFl^gr^.

## Conclusion

The study confirmed the association of the changes in clinical findings, whole blood picture, serum testosterone, serum cortisol, and semen analysis, with OnlyBp^gr^ and BpFl^gr^. These changes were more prominent in BpFl^gr^ than in OnlyBp^gr^. These changes were also more evident in BpFl^gr^ and OnlyBp^gr^ than in Cont^gr^. Skin scraping test results revealed a higher severity of live ticks’ infestation in BpFl^gr^ than in OnlyBp^gr^ because, unlike OnlyBp^gr^, all camels in BpFl^gr^ (*n* = 10) were suffering from live ticks’ infestation. The present work also concluded the higher efficacy of histopathology of testicular tissues in male camels as a diagnostic tool for adult filaria in balanoposthitis-affected male camels than blood smear because all cases of camel filariasis in the current work were negative for microfilaria on microscopic examination of diurnal blood smear as well as testicular histopathology revealed detection of adult filaria in all camel filariasis associated with balanoposthitis. Strong correlations were demonstrated between serum testosterone, serum cortisol, and semen analysis results. Positive correlations were reported between serum testosterone levels and sperm vitality percentages. However, negative correlations were stated between serum testosterone and each of serum cortisol and sperm abnormalities either in Cont^gr^, OnlyBp^gr^ or BpFl^gr^. Serum cortisol concentrations were positively correlated with sperm abnormalities percentages. However, they were negatively correlated with sperm vitality percentages in Cont^gr^, OnlyBp^gr,^ or BpFl^gr^. Negative correlations were observed between sperm vitality percentages and sperm abnormalities percentages either in Cont^gr^, OnlyBp^gr,^ or BpFl^gr^.

## Data Availability

The datasets used and/or analyzed during the current study are available from the corresponding author upon reasonable request. Data was also available after publishing in this journal.

## Abbreviations

BpFl^gr^: balanoposthitis-filariasis infected male camels group
*D. evansi*: (*Dipetalonema evansi*)
DLC: differential leukocytic count
Hb: hemoglobin
OnlyBp^gr^: only balanoposthitis without filariasis male camels group
MCS: manure condition score
MDS: manure digestion score
PCV: packed cell volume
RBCs: red blood corpuscles
RFS: rumen filling score
TLC: total leucocytic count
OnlyBp^gr^: only balanoposthitis without filariasis male camels group
